# Kinetic, Isotherm and Thermodynamic Aspects of Zn^2+^ Biosorption by *Spirulina platensis*: Optimization of Process Variables by Response Surface Methodology

**DOI:** 10.3390/life12040585

**Published:** 2022-04-14

**Authors:** Nada K. Alharbi, Mayasar I. Al-Zaban, Fawziah M. Albarakaty, Sayed F. Abdelwahab, Sedky H. A. Hassan, Mustafa A. Fawzy

**Affiliations:** 1 Department of Biology, College of Science, Princess Nourah bint Abdulrahman University, P.O. Box 84428, Riyadh 11671, Saudi Arabia; nkalharbi@pnu.edu.sa (N.K.A.); mialzaban@pnu.edu.sa (M.I.A.-Z.); 2 Department of Biology, Faculty of Applied Science, Umm Al-Qura University, P.O. Box 715, Makkah Al Mukarramah 21955, Saudi Arabia; fmbarakati@uqu.edu.sa; 3 Department of Pharmaceutics and Industrial Pharmacy, College of Pharmacy, Taif University, P.O. Box 11099, Taif 21944, Saudi Arabia; s.fekry@tu.edu.sa; 4 Department of Biology, College of Science, Sultan Qaboos University, Muscat 123, Oman; s.hassan@squ.edu.om; 5 Department of Botany and Microbiology, Faculty of Science, New Valley University, El-Kharga 72511, Egypt; 6 Biology Department, Faculty of Science, Taif University, P.O. Box 11099, Taif 21944, Saudi Arabia

**Keywords:** biosorption, equilibrium isotherm models, pseudo-second order, response surface methodology, *Spirulina platensis*, zinc

## Abstract

The aim of this study was to assess the efficiency of *Spirulina platensis* for removing Zn^2+^ ions from the aqueous solutions. The optimized conditions of 4.48 g/L algal dose, pH of 6.62 and initial zinc concentration of 29.72 mg/L obtained by response surface methodology were employed for Zn^2+^ biosorption by *S. platensis* and up to 97.90% Zn^2+^ was removed, showing that there is a favorable harmony between the experimental data and model predictions. Different kinetic and equilibrium models were used to characterize the biosorption manner of *Spirulina* as a biosorbent. The kinetic manner of Zn^2+^ biosorption was well characterized by the pseudo-second-order, implying that the adsorption process is chemical in nature. The Langmuir and Dubinin–Radushkevich isotherm models were best fit to the equilibrium data. The maximum adsorption capacity of the Langmuir monolayer was 50.7 mg/g. Furthermore, the thermodynamic analysis revealed that Zn^2+^ biosorption was endothermic, spontaneous and feasible. As a result of biosorption process, FTIR, SEM, and EDX investigations indicated noticeable alterations in the algal biomass’s properties. Therefore, the dried *Spirulina* biomass has been shown to be cost-effective and efficient for removing the heavy metals, particularly zinc ions from wastewater, and the method is practicable, and environmentally acceptable.

## 1. Introduction

Water consumption has risen dramatically as a result of the technological advancement, extraordinary industrial development, and urbanization, resulting in massive amounts of toxic effluent [1]. Wastewater can include a variety of heavy metals that pollute water where marine species exist, such as fish consumed by human. As these heavy metals bioaccumulate via the food chain, they endanger not just aquatic life, but also human health. Nickel, lead, copper, chromium, cadmium, arsenic and zinc are the most common pollutants in water [2].

Zinc is found in a variety of metal proteins and enzymes, and is involved in various biological processes that are necessary for organisms to grow and develop normally. On the other hand, overabundance of it in the water can cause DNA damage and oxidative stress, as well as growth and reproductive problems [3,4]. Additionally, the existence of zinc ions in wastewater is a threat to the aquatic organisms, as well as a source of several health hazards for humans [4], and consequently, it is critical to remove it from water supplies [5].

The removal of heavy metals was achieved using traditional treatment methods such as membrane separation, filtration, electrochemical treatment, ion exchange and chemical precipitation [6]. These methods, on the other hand, are economically unviable and ineffective [7]. Biosorption has lately developed as a possible alternative to traditional heavy metal removal methods [8]. Biosorption is an efficient, eco-friendly and low-cost method that uses a biomaterial as a source. The main benefit of biosorption methods is their ability to decrease high heavy metal levels to lower concentrations. As a result, biomaterials such as fungi, bacteria, algae, and their by-products can be employed as biosorbents [9,10,11,12,13].

Heavy metal removal applications have attracted a lot of interest in algae-based biosorbents, which are inexpensive, have a great surface area and a high binding affinity [14]. Several studies have shown that the surface of organisms include different functional charged groups that are important for binding of heavy metals, such as amine, hydroxyl, carboxyl, sulfate groups, etc., making these organisms attractive for low-cost treatment of wastewater [15,16]. Several variables influence the bioremoval of heavy metals, such as the biomass dosage, concentration of metal ion, pH of solution, and contact time.

Lately, response surface methodology (RSM) has been widespread as a statistical tool for rational experimental design and optimization of process variables. It is a set of statistical and mathematical approaches for determining the importance of a number of influencing parameters in the optimum behavior, especially when complex correlations are present [17]. It does not need extra chemicals consumption for each factor, nor is it particularly labor-intensive, costly, increased experiments number, or time-consuming, in contrast to the conventional approaches that have been achieved by determining the effect of one parameter at a time [18].

In this study, response surface methodology was combined with Box−Behnken design (BBD) to assess the interactive impacts and determine the best conditions for some parameters such as algal dosage, pH of solution, and initial Zn^2+^ concentration for obtaining the maximum removal of zinc ions from aqueous solutions using *Arthrospira* (*Spirulina*) *platensis* biomass. The kinetic experimental data were fitted to pseudo-first, -second order, and intra-particle diffusion models, while the isotherm results were fitted to Langmuir, Freundlich, and Dubinin–Radushkevick models to evaluate the mechanism of adsorption process onto the surface of *Spirulina* biomass. Furthermore, thermodynamic modeling was used to investigate the feasibility of the biosorption process. FTIR and SEM/EDX investigations were used to examine the algal biomass before and after biosorption process.

## 2. Materials and Methods

### 2.1. Cultivation and Preparation of Biosorbent

The cyanobacterial alga *Arthrospira* (*Spirulina*) *platensis* Gomont was isolated from the faculty of agriculture farm at Assiut University (Egypt) and identified according to Prescott [19]. The algal species was cultivated in a 5 L of Zarrouk’s medium [20], incubated at 30 °C under continuous illumination of 48.4 µmole m^−2^ s^−1^ and the pH of the medium was adjusted to 9. Centrifugation at 6000 rpm for 10 min was used to collect the algal biomass, which was then dried at 60 °C and ground into a fine powder for using in the biosorption tests.

### 2.2. Preparation of Zinc Solutions

About 2.084 g ZnCl_2_ was dissolved in 1 L distilled water for preparing the stock solution of Zn^2+^ ions.

### 2.3. Characterization of the Spirulina Biomass

The morphology of the algal biomass surface was examined by scanning electron microscope (SEM) (JEOL JSM-6510 L.V operated at 30 KV) combined with energy-dispersive X-Ray analysis (EDX) (JEOL JEM-2100 (HRTEM), as well as Fourier transform infrared (FT-IR) spectroscopy (Thermo Fisher Scientific model FT-IR is 10, Waltham, MA, USA). This was performed to better understand the morphology and properties of the algal surface, as well as to investigate the correlation between structural properties of the biosorbent and adsorption behavior.

### 2.4. Batch Studies of Biosorption

#### 2.4.1. Influence of Individual Variables

The impact of various parameters on the removal efficiency of *S. platensis* was studied using batch sorption tests. To examine how varied contact time (0–180 min) affected the biosorption of Zn^2+^ ions, approximately 3 gL^−1^ of the dried biomass was added to 60 mg/L of initial metal concentration at pH 6.

The effect of initial ion concentrations on Zn^2+^ ion removal was examined under the conditions of 3 gL^−1^ of algal dosage, pH 6 and contact time of 60 min with varied initial Zn^2+^ concentration (20–100 mg/L). The impact of temperature was performed at different temperature values of 25, 35 and 45 °C, under the conditions of 3 gL^−1^ of algal dosage, initial Zn^2+^ concentration of 60 mg/L, pH 6 and contact time of 60 min. The pH of solution was adjusted with 0.1 M NaOH and/or H_2_SO_4_. For all tests, flasks containing 100 mL of biosorption samples were shaken at 180 rpm and 25 °C. The algal biomass was then centrifuged at 4000 rpm for 5 min to separate it from the aqueous solutions, and Zn^2+^ concentration in supernatant was analyzed using Inductively Coupled Plasma-Optical Emission Spectrometer (ICP-OES) model Perkin Elmer Optima 2000 DV. The efficiency of Zn^2+^ elimination was then calculated from the following Equation (1):

(1) $$\left(\%\right)\;\mathrm{Removal}\;\mathrm{efficiency}=\frac{C_{i}-C_{eq}}{C_{i}}\times 100\;$$

where *C_i_* (mg/L): the initial concentration of Zn^2+^ ion, and *C_eq_*: the equilibrium Zn^2+^ concentration left in the solution.

The amount of Zn^2+^ ion adsorbed on the algal surface at equilibrium (*q_e_*; mgg^−1^) was calculated from the Equation (2):

(2) $$q_{e}=\frac{V\left(C_{i}-C_{eq}\right)}{W\;}\;\;\;\;\;\;\;\;\;\;\;\;\;$$

where *V*: is the volume of Zn^2+^ solution (mL), and *W*: is the weight of algal biomass (g).

#### 2.4.2. Optimization of Process Variables

In order to evaluate the optimal conditions for maximizing the biosorption of Zn^2+^ ions onto *Spirulina* biomass, the response surface methodology was used. The design includes three parameters; algal dosage (A; 1–5 g/L), pH (B; 3–7), and initial Zn^2+^ concentration (20–60 mg/L), with the response being the percentage of removal efficiency. Each parameter was coded with one of three levels: −1 (low), 0 (medium), and +1 (high).

Table 1 shows the parameters’ coded form, as well as their range and levels. The biosorption experiment was designed with Box–Behnken Design, which resulted in a total of 17 runs. The biosorption tests were performed with a constant temperature of 25 °C, a contact time of 60 min and shaking at 180 rpm, and the residual Zn^2+^ concentration was then calculated as previously described. The second order polynomial quadratic model for predicting optimal conditions may be represented by the Equation (3):

Y = βₒ + ∑ β_i_X_i_ + ∑ β_ii_X_i_^2^ + ∑ β_ij_X_i_X_j_ + ε (3)

where Y: represents the response, βₒ: the constant coefficient, β_i_, β_ii_ and β_ij_: the linear, quadratic, and second-order interaction impacts, respectively; X_i_ and X_j_: the non-coded variables, and ε: the error.

For evaluating the interaction between the independent factors and response, the data were analyzed using the application of Design-Expert 7.0, which included ANOVA and three-dimensional surface plots. The determination coefficient (*R*^2^) was used to represent the quality of the fit of the quadratic model, and the *F*-test was used to evaluate the statistical significance.

#### 2.4.3. Kinetics Model Analysis

Biosorption kinetics were studied at 25 °C with a time range of 0–180 min and constant conditions of algal dose of 3 g/L, pH 6, and initial metal concentration of 60 mg/L. The quantity of adsorbed Zn^2+^ ions onto the algal biomass at equilibrium (*q_e_*) and at any time *t* (*q_t_*) was then computed to estimate the biosorption kinetics. The pseudo-first, -second-order, and intra-particle diffusion models were utilized to evaluate the mechanism and rate of the biosorption process.

#### 2.4.4. Isotherm Model Analysis

The isotherm of the biosorption process was investigated at 25 °C with various initial concentrations of Zn^2+^ ions (20, 40, 60, 80, 100 mg/L) and constant conditions of 3 g/L algal dose, pH 6, and contact time of 60 min. The adsorption isotherm was then determined by calculating the quantity of adsorbed zinc ions at equilibrium (*q_e_*), equilibrium Zn^2+^ ion concentration (*C_eq_*), and specific biosorption (*C_eq_/q_e_*). Three isotherm models including Langmuir, Freundlich, and Dubinin–Radushkevich were used to describe the biosorption isotherm.

#### 2.4.5. Thermodynamic Model Analysis

The purpose of this analysis was to determine the feasibility of the biosorption process. The thermodynamic parameters were achieved from the experiments at various temperatures (298, 308 and 318 K) with constant conditions of 3 g/L algal dose, pH 6, initial metal concentration of 60 mg/L and contact time of 60 min.

## 3. Results and Discussion

### 3.1. Characterization of S. platensis Biomass

#### 3.1.1. FT-IR Analysis

**Figure 1:**
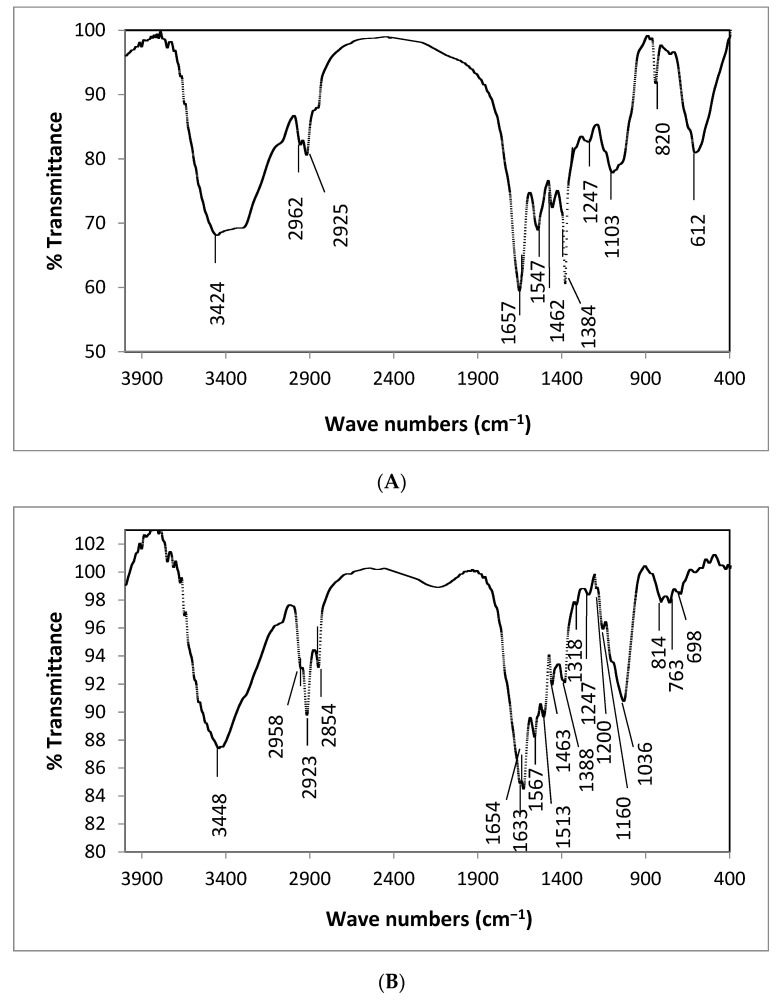
FT-IR spectra of algal biomass (**A**) before, and (**B**) after Zn^2+^ ions biosorption.

The functional groups of the algal biomass that responsible for the biosorption process were identified using FT-IR analysis. Figure 1 shows the FT-IR data before (A) and after (B) biosorption of Zn^2+^ ions onto the *Spirulina* biomass. Several functional groups were found on the surface of algal biomass, according to the FT-IR spectrum data. The wide peak at 3424–3448 cm^−1^ was attributed to the –OH bending vibration of polysaccharides and N–H of the amine groups [21]. The absorption peaks at 2962–2958 cm^−1^ and 2925–2923 cm^−1^ were ascribed to the –CH of methyl (–CH_3_) and methylene (–CH_2_) groups [22]. The stretching vibration of C–H, which indicate the existence of carboxylic groups, are represented by the new peak found only after Zn^2+^ biosorption at 2854 cm^−1^ [23]. In addition, after biosorption of Zn^2+^ ions by *Spirulina* biomass, a prominent absorption band at 1742 cm^−1^ was found, which might be due to the stretching vibration of C=O in ester or –COOH groups (Figure 1B) [24]. Slight shifts recorded after Zn^2+^ biosorption indicating that –OH, –COOH, and N–H groups on the surface of *Spirulina* were responsible for the process of biosorption via complexation mechanism. The bands around 1657–1654 cm^−1^ and 1547–1567 cm^−1^ were associated with the amide I and amide II stretching of proteins, respectively [25]. The new peak at 1633 cm^−1^ appeared only in the algal biomass after biosorption of Zn^2+^ ions, suggested that the stretching vibration of C=N group involved in the biosorption process [26]. Furthermore, after Zn^2+^ biosorption, carboxylate ion peaks were found only on the surface of *Spirulina* biomass at 1513 cm^−1^ and 1160 cm^−1^ [27]. The adsorption peaks at 1462–1463 cm^−1^ were interrelated to stretching of CH_2_ of aliphatic, whereas the peaks around 1384–1388 cm^−1^ was attributed to the C=O bending vibration of carboxyl group [28]. The P–O–C stretching vibration of the organic phosphate groups is responsible for the band appeared at 1036 cm^−1^ on the algal surface treated with zinc ions [29]. The phosphodiester P=O stretching vibration was responsible for the protein spectra appeared at 1247 cm^−1^ following Zn^2+^ biosorption. Furthermore, the shifting of the biosorption band from 820 cm^−1^ before biosorption to 814 cm^−1^ after Zn^2+^ biosorption reveals the binding of Zn^2+^ ions to the amine group on the surface of *Spirulina* biomass [30]. The existence of several functional groups on the algal biomass surface including carboxyl, hydroxyl, amine, amide, phosphate, methylene and methyl groups, proposes that the alga has a tendency for biosorbing zinc ions. These functional groups play a significant role in heavy metal biosorption by producing complexes and ion exchange as a result of metal ions’ attraction for the functional groups. Other researchers who studied the metal ion biosorption onto *Spirulina* sp. found similar results. In this regard, Ferreira et al. [31] emphasized the importance of carboxyl functional groups in zinc, lead and nickel ions biosorption onto the biomass of *Spirulina platensis*. Rezaei [32] also revealed that amino, hydroxyl, carbonyl and carboxylic groups were involved in Cr^6+^ ions biosorption by *Spirulina* biomass.

#### 3.1.2. SEM/EDX Examination

**Figure 2:**
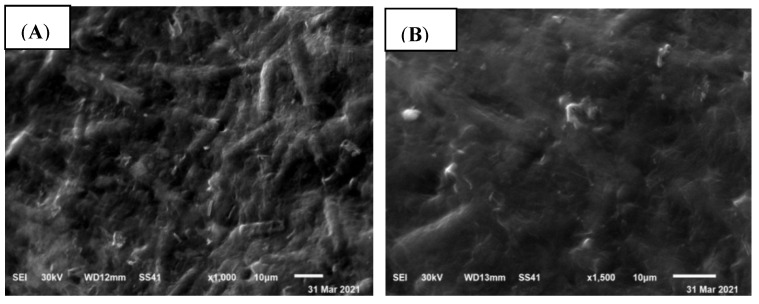
SEM images of algal biomass (**A**) before, and (**B**) after Zn^2+^ ions biosorption.

Scanning electron microscopy (SEM) was used to examine the surface morphology of *Spirulina* biomass before and after biosorption process (Figure 2). The cells had a non-uniform and rough surface with holes prior to zinc biosorption, showing that there is a significant potential for biosorption of Zn^2+^ ion (Figure 2A). The cell surface was flat following the biosorption process, with some aggregations clustered on the algal surface (Figure 2B). This might be due to accumulation of Zn^2+^ ion onto the algal surface, which is connected to the biosorbent’s functional groups. In this regard, Babu et al. [33] used scanning electron microscopy to examine the *Spirulina platensis* surface before and after zinc biosorption and revealed that Zn^2+^ ions precipitated at the algal surface.

**Figure 3:**
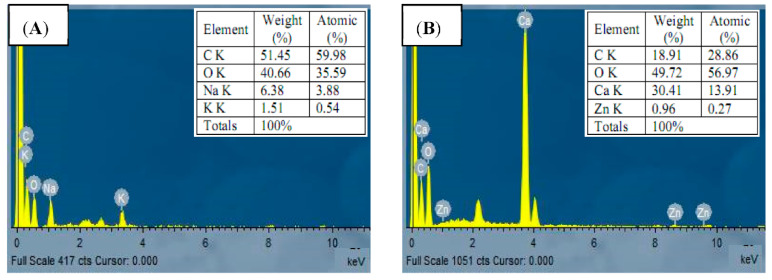
EDX image of algal biomass (**A**) before, and (**B**) after Zn^2+^ ions biosorption.

The analysis of elemental composition of *Spirulina* biomass before and after zinc ions biosorption was studied by energy-dispersive X-Ray analysis (EDX) (Figure 3). Figure 3A depicted a significant carbon and oxygen content with minor levels of sodium and potassium. However, zinc-loaded *Spirulina* biomass displayed an additional peak of Zn^2+^ ion, in addition to carbon, oxygen, and calcium, indicating that the algal biomass is responsible for the biosorption process (Figure 3B). The weight composition (%) of oxygen and carbon in zinc-loaded *Spirulina* biomass was significantly changed, proposing that the hydroxyl and carboxylic groups on the algal surface are important for the biosorption of Zn^2+^ ions. Furthermore, the exchange and disappearance of some elements after biosorption process suggested that biosorption of Zn^2+^ ions was caused by ion exchange. In this respect, Ahmad et al. [10] reported that when *Chlorella vulgaris* biomass was treated with zinc ions, the characteristic zinc peak was formed. SEM/EDX examination of *Spirulina* sp. after biosorption demonstrated a rise in Zn^2+^, Mn^2+^, Cu^2+^ and Co^2+^ ions employed in biosorption, according to Dmytryk et al. [34].

### 3.2. Impact of Contact Time

Contact time influences the equilibrium kinetics, confirms the adsorption process stability, and provides an estimate of the overall cost when developing a large-scale sorption system [35].

**Figure 4:**
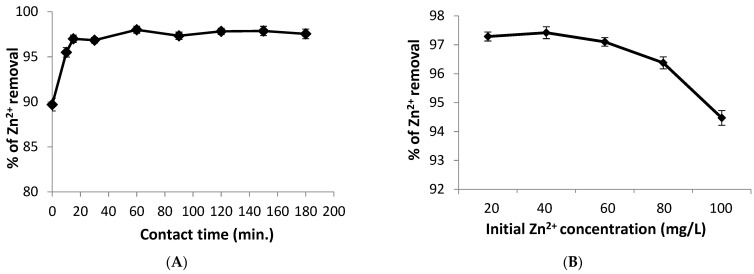
Effect of (**A**) Contact time, and (**B**) Initial Zn^2+^ ions concentration on removal efficiency.

The effect of contact time on Zn^2+^ biosorption onto *S. platensis* was investigated using a range of time (0–180 min) and constant biosorption conditions of 3 g/L algal dose, pH 6, and initial ion concentration of 60 mg/L at 25 °C, as shown in Figure 4A. The data showed that the Zn^2+^ removal efficiency increased rapidly to 97% with time of 15 min. The biosorption of Zn^2+^ is unaffected by increasing the contact time, implying that the equilibrium of biosorption may be achieved within 60 min with 98.0% Zn^2+^ elimination. This is because the biosorbent initially had plenty of active sites and higher surface area accessible to absorb zinc ions. While, the adsorption effectiveness reduces when these sites are gradually occupied, leading to decreasing biosorption process [36].

### 3.3. Impact of Different Adsorbate Concentrations

The initial metal concentration is found to be a crucial factor in reducing the adsorbate mass transfer resistance between the solid adsorbent and solution. In this study, the removal percentage of Zn^2+^ ions onto the *Spirulina* biomass was decreases from 97.42% to 94.47% with increasing the initial ion concentration from 40 to 100 mg/L (Figure 4B). The high removal percentage at lower concentration of zinc is because of the plenty of binding sites on the surface of algal biomass. However, at higher ion concentrations, the metal uptake is reduced or almost constant due to the lack of the surface active sites for further adsorption. Several researchers reached to the same conclusion [37,38], finding that raising metal concentrations lowered biosorption of ions by the algal biomass. Thus, the surface saturation by metal ions was shown to be dependent on the initial concentrations of heavy metals [39].

### 3.4. Optimization of Zinc Ions Removal Using Response Surface Methodology

For optimizing the zinc removal efficiency by *Spirulina* biomass, response surface methodology (RSM) and Box–Behnken design (BBD) were applied. The interaction impact of the three parameters such as (A) algal dose, (B) pH, and (C) initial zinc concentration at three levels on the biosorption of Zn^2+^ ions was investigated with 17 runs produced from BBD.

Zinc removal efficiency by *S. platensis* was determined to be between 70.0 and 99.33% (Table 1). The actual uptake of Zn^2+^ ions was likewise found to be rather similar to the expected biosorption capacity by *Spirulina* biomass.

According to the BBD, a quadratic polynomial equation was found for determining the optimal value. The equation relating biosorption efficiency and input parameters in coded terms in the empirical model is presented as Equation (4) after backward exclusion for the insignificant variables.

% removal (Zn) = 86.72+ 4.82A + 6.10B − 4.23 C − 4.54AB + 7.65AC + 4.65BC + 5.17B^2^ (4)

where A, B, and C: represent the coded values for the independent parameters; algal dosage (A; g/L), pH (B), and initial Zn^2+^ concentration (C; mg/L).

The ANOVA is used to assess the model’s suitability, and the results for zinc ion removal are reported in Table 2. The *p* value, *F* value, determination coefficient (*R*^2^), and the data of the lack of fit test may all be used to assess the significance of the quadratic model [40]. The regression was statistically significant for Zn^2+^ ions, with *F* value of 6.71 and *p* > *F* value of 0.005.

The regression equation effectively describes the response since the model’s lack of fit was determined to be insignificant [41].

The quadratic model was significant and adequate to depict the actual interaction between the independent variables and the response, as evidenced by the high determination coefficient (*R*^2^ = 0.89). Additionally, the adjusted *R*^2^ (0.81) was in reasonable agreement with the expected *R*^2^ (0.70; Table 2).

Simultaneously, a low variation coefficient (CV = 5.5%) shows that the experiments were performed with accuracy and dependability [42].

The adequate precision value of the quadratic model was 8.4, indicating that there was a good signal, and the polynomial model could be utilized to explore the design space. In this respect, a signal-to-noise ratio greater than 4 is desirable, according to Yavari et al. [43].

### 3.5. The Interactive Impact of Process Parameters and 3-D Response Surface Plots

**Figure 5:**
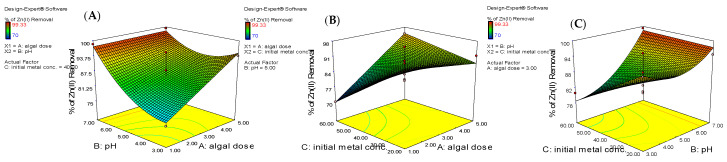
Response surface plots showing the influence of interactive parameters on Zn^2+^ ions removal efficiency (**A**) algal dosage and pH, (**B**) algal dosage and initial Zn^2+^ concentration, and (**C**) pH and initial Zn^2+^ concentration.

The interactive impact of the independent variables, such as algal dose, pH, and ion concentration, on the removal % of Zn^2+^ ions are shown as 3-D surface plots (Figure 5). Figure 5A depicts a 3-D plot of the mutual impact of biosorbent dosage and pH at a fixed concentration of zinc ion (40 mg/L). When the algal dose was raised from 1.0 to 5.0 g/L and the pH from 3.0 to 7.0, the efficiency of zinc removal improved. According to the response variable’s coded equation, the influence of algal dose (A) and pH (B) on the efficiency of zinc removal by *Spirulina* biomass is positive, with coefficients of 4.82 and 6.10, respectively (Equation (4); Table 3). Furthermore, the ANOVA analysis demonstrates that the algal dose and pH have significant impacts on biosorption efficiency, with *p* values of 0.038 and 0.007, respectively (Table 3). The highest removal of zinc ions occurs at pH 7 and a 5.0 g/L algal dose. The biosorbent dosage affects the quantity of active functional groups for the biosorption process. Increasing removal of zinc ions at higher algal dosage may be due to existence of more binding sites on the algal surface that is easily available for biosorption [44]. This indicates that the maximum removal % is achieved by increasing the algal dose and pH at the same time.

Figure 5B illustrates the removal percentage of zinc ions as a function of algal dose and initial zinc concentration. The removal percentage of zinc decreased as the initial concentration of Zn^2+^ ions increased. This was supported by the results of ANOVA, which revealed that the zinc concentration had a negative impact on the efficiency of zinc removal (−4.23; Table 3). In addition, the initial zinc concentrations have greater impact than the algal dose. It also demonstrated that, at algal dosage of 5 g/L and initial zinc concentration of 20 mg/L, the highest removal percentage of zinc ions was reached. The limited binding sites on the surface algal biomass at high metal concentrations may explain a reduction in removal percentage when the initial metal concentration is increased [45].

Figure 5C depicts the influence of pH and initial zinc concentrations on the efficiency of zinc elimination. At a fixed algal dosage of 3.0 g/L, zinc removal percentage reduced when the initial zinc concentration increased from 20 to 60 mg/L and enhanced when pH increased from 3.0 to 7.0. Changing the pH of a solution can alter the surface charge of a biosorbent. So, the point of zero charge for the algal biomass was determined [46]. It was found that the algal biomass surface has pH_PZC_ of 6.5. Furthermore, the removal percentage of zinc ion by *S. platensis* biomass increased with rising pH values (pH > pH_PZC_). This is attributed to the fact that the algal surface acquires a positive charge at pH values < pH_PZC_ due to the existence of positively charged hydrogen ion in the aqueous solution, making it difficult for metal ions with a positive charge to approach the functional groups on the algal surface due to electrostatic repulsion [47]. On the other hand, the H^+^ concentration drops when pH rises (pH > pH_PZC_), and the surface charge of the biosorbent becomes negative. As a result, the electrostatic interaction force between the *S. platensis* biomass and Zn^2+^ ion rises, resulting in a higher removal percentage (≈98%) onto the algal surface at pH 7 [48].

### 3.6. Validation Experiment of the Optimized Conditions

The experimental validation was carried out to assess the optimal values of process parameters for maximizing the biosorption of Zn^2+^ ions onto the *Spirulina* biomass. The optimum biosorption conditions of algal dose (4.48 g/L), pH (6.62), and initial zinc concentration (29.72 mg/L) were evaluated at a contact time of 60 min and 25 °C. Under these conditions, the experimental and predicted removal percentage of 97.90 and 99.45%, respectively are in a good accordance, suggesting that the quadratic model might be used to accurately explain the interaction between the independent variables and response.

### 3.7. Adsorption Kinetics

When using adsorption to remove a heavy metal, it’s important to evaluate the process kinetics, which can help the process design in real-life conditions. Several studies have been presented kinetics models based on the concentration of metal adsorbed on the adsorbent as a function of time [49]. The pseudo-first order, pseudo-second order, and intra-particle diffusion models have been used in the current investigation to assess the mechanism and the rate of biosorption process.

Adsorption of one adsorbate molecule onto one active site is suggested by the pseudo-first order model [50]. It is expressed as follows:

(5) $$Log\;\left(q_{e}-q_{t}\right)=Log\;q_{e}-\frac{K_{1}t}{2.303}\;$$

where *q_e_* and *q_t_* (mg/g): represent the quantity of adsorbate uptake sorbed on the biosorent surface at equilibrium and any time (*t*), respectively, and *k*_1_ (1/min): represents the rate constant of pseudo-first order equation.

**Figure 6:**
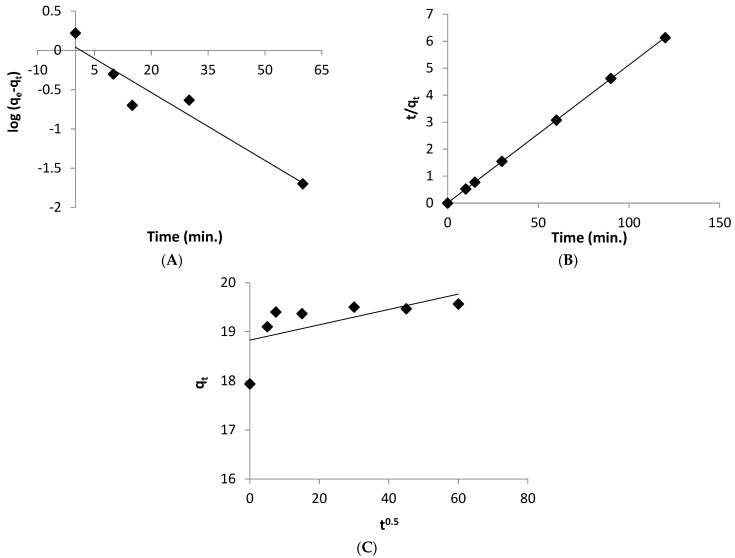
Different kinetic model plots (**A**) Pseudo-first order, (**B**) Pseudo-second order, and (**C**) Intraparticle diffusion model.

The values of *q_e_* and *k*_1_ were determined from the intercept and slope of *log* (*q_e_* − *q_t_*) versus *t* plot, respectively (Figure 6A), and the pseudo-first order parameters are listed in Table 4.

Pseudo-second order model suggests that one adsorbate molecule is sorbed onto two binding sites of the surface of biosorbent [51]. It is expressed by the following Equation (6):

(6) $$\frac{t}{q_{t}}=\frac{1}{K_{2}q_{e}^{2}}+\frac{t}{q_{e}}\;\;\;$$

where *k*_2_ (g mg^−1^ min^−1^): the rate constant of pseudo-second order model. The values of *q_e_* and *k*_2_ were estimated from the slope and intercept of *t*/*q_t_* vs. *t* plot, respectively (Table 4 and Figure 6B).

Another model for calculating the rate of adsorption process is intra-particle diffusion. If a plot of *q_t_* versus *t*^0.5^ provides a straight line, the intra-particle diffusion is the rate-controlling step in the adsorption process [52]. This model is represented as follows:

(7) $$q_{t}=K_{i}t^{0.5\;}+C_{i}$$

where *k_i_* (mgg^−1^ min^−0.5^): represents the rate constant of intra-particle diffusion, and *C_i_* (mgg^−1^): is a constant which give an impression about the boundary layer thickness on the surface of biosorbent. The values of *k_i_* and *C_i_* are calculated using the slope and intercept of the linear plot of *q_t_* against *t*^0.5^ (Figure 6C).

The kinetic parameters for the biosorption of zinc ions by *Spirulina* biomass are presented in Table 4. The results indicated that the value of determination coefficient (*R*^2^) achieved by the pseudo-second order model (0.999; Figure 6A) is greater than that obtained from the pseudo-first order model (0.816; Figure 6B) and the intra-particle diffusion model (0.382; Figure 6C). Furthermore, the adsorption capacity calculated from second-order kinetic model (*q_e_* cal. = 19.57 mg g^−1^) agrees well with that determined by the experiment (*q_e_*exp. = 19.60 mg g^−1^) compared to the data of psudo-first order model.

These findings validate the model’s underlying premise that adsorption is connected to chemisorption by indicating that the kinetics of Zn^2+^ ions adsorption onto *Spirulina* biomass follow the pseudo-second order model [53]. The binding sites in the surface of *Spirulina* biomass, which include rich groups such as amide and hydroxyl, as indicated from FT-IR data give a good possibility for the metal ion chemisorption. As shown in Table 4, the pseudo-second order rate constant (0.29 g mg^−1^ min^−1^) was greater than the rate constant of the first-order kinetic (0.066 min^−1^) for the zinc uptake by *Spirulina* biomass. This finding is in line with the fact that Zn^2+^ ions require less time to achieve adsorption equilibrium.

Additionally, when the kinetics data were evaluated by the intra-particle diffusion model, it was detected that the plot did not pass through the origin, showing that the intra-particle diffusion model was not the only rate-controlling step in the biosorption process (Figure 6C) [54]. It was indicated also that the greater the intercept (*C_i_*, 18.83 mg/g), the larger the contribution of surface biosorption in the rate-controlling step through film diffusion. Thus, the film diffusion and intra-particle diffusion worked concomitantly during the biosorption process. In addition, the rate constant of intra-particle diffusion (*K_i_*) was high, showing that the *Spirulina* biomass had a higher biosorption capacity and better bonding between the Zn^2+^ ions and biosorbent [21].

### 3.8. Adsorption Isotherm

**Figure 7:**
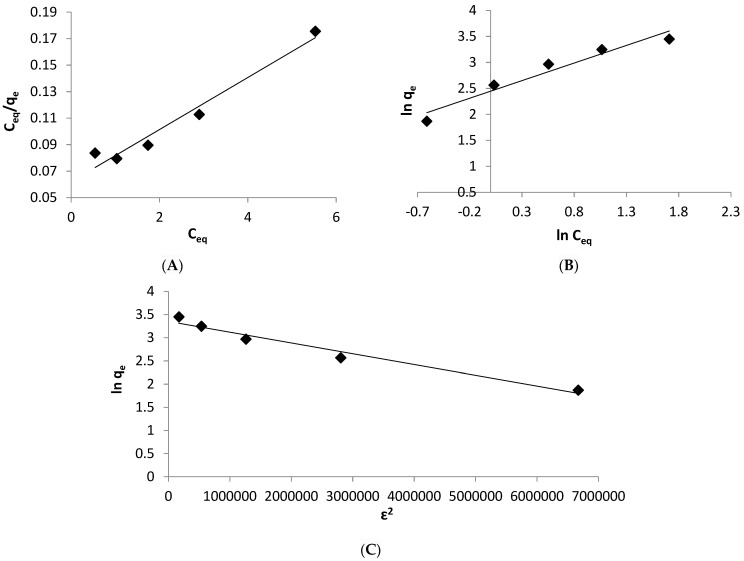
Different isotherm model plots (**A**) Langmuir, (**B**) Freundlich, and (**C**) Dubinin–Radushkevick model.

Adsorption isotherm models may be used to explain the equilibrium data isotherm for the biosorption of Zn^2+^ ions by *Spirulina* biomass. They were described using the Langmuir, Freundlich, and Dubinin–Radushkevick (D–R) adsorption models. The Langmuir model explains the homogeneous surface of adsorption sites, as well as the lack of interactions between adsorbate ions. The following Equation (8) was used to determine the Langmuir model:

(8) $$\frac{C_{eq}}{q_{e}}=\frac{1}{q_{max}b}+\frac{C_{eq}}{q_{max}}\;\;\;\;\;\;\;\;$$

where the maximum sorption capacity *q_max_* (mg/g) and the Langmuir constant *b* (L/mg) may be derived from the slope and intercept of the plot of *C_eq_/q_e_* against *C_eq_*, respectively (Figure 7A).

The Langmuir isotherm constant (*b*), which describes the favorable adsorption process, is a useful tool in estimating the dimensionless constant separation factor (*R_L_*) (Equation (9)).

(9) $$R_{L}=1/\left(1+bC_{o}\right)$$

where *C_o_*: represents the initial Zn^2+^ ions concentration.

The nature of the biosorption process is showed by the *R_L_* value, which might be favorable (0 < *R_L_*< 1), unfavorable (*R_L_* > 1), linear (*R_L_* = 1) or irreversible (*R_L_* = 0) [55].

The Freundlich model is most commonly used to represent the adsorption on heterogeneous adsorbents. It is explained as follows:

(10) $$Ln\;q_{e}=Ln\;K_{f}+\frac{1}{n}\;Ln\;C_{eq}$$

where *K_f_*: is the adsorption capacity, and *n*: is adsorption affinity, which may be determined from the intercept and slope, respectively, of the linear plot of ln *q_e_* and ln *C_eq_* (Figure 7B).

The energy of the biosorption process, whether chemical or physical, was investigated using the Dubinin-Radushkevich model [56]. The following Equation (11) can be used to illustrate this model:

(11) $$Lnq_{e}=lnq_{0}-\beta \varepsilon^{2}\;$$

(12) $$\varepsilon =RTln(1+\frac{1}{C_{eq}})$$

(13) $$E=\sqrt{1/2}\beta$$

where *q_o_* (mg/g): is theoretical saturation capacity of the adsorbent, *β*: is the energy of sorption, *ε*: is the polyani potential, *R*: represents the universal gas constant (8.314 kJ mol^−1^), and *T* (K): is the absolute temperature.

The *R*^2^ values and other parameters of Langmuir, Freundlich, and Dubinin-Radushkevich models are tabulated in Table 5.

The results indicated that the Langmuir and Freundlich isotherm models fit well the biosorption data of zinc ions elimination onto the *Spirulina* biomass, as evidenced by high *R*^2^ values (Table 5). However, the Langmuir model appears to be more appropriate than the Freundlich model, as the *R*^2^ value of Langmuir model is somewhat greater than that evaluated by the Freundlich model. The best fit to the Langmuir isotherm model indicates that the adsorption sites on the surface of algal biomass are homogeneous, providing a monolayer coverage of metal ions on the surface of *Spirulina*. In this respect, Babu et al. [33] investigated the biosorption of Zn^2+^ onto the biomass of *Arthrospira platensis* and found similar findings.

The maximum biosorption capacity (*q_max_*) of zinc ions onto the biomass of *Spirulina platensis* was 50.7 mg/g indicating that, the algal biomass could be used as a potential biosorbent for the elimination of zinc ions from the aqueous solutions. The calculated maximal absorption capacity was greater than the experimentally determined value (31.5 mg/g), implying that the biosorption of Zn^2+^ onto the biomass of *Spirulina* might occur at higher concentrations of zinc. Furthermore, the values of *R_L_* (0.03–0.12) indicate that the biomass of *Spirulina platensis* is capable of biosorbing Zn^2+^ ions and the biosorption process is favorable. The value of *1/n* (0.68; Table 5) attained from the Freundlich isotherm model between 0 and 1, indicates a greater zinc/biosorbent interaction, and the Zn^2+^ ions are preferentially biosorbed onto the biomass of *Spirulina platensis* [3].

The biosorption data was also examined by the D–R model to assess the nature of the biosorption process as chemical or physical. The sorption energy for the ion exchange or chemical biosorption varies from 8 to 16 kJ mol^−1^, whereas the physical adsorption requires less than 8 kJ mol^−1^ [44]. The value of *E* in this investigation was larger than 8 kJ mol^−1^ (15.8 KJ mol^−1^; Table 5; Figure 7C), indicating that a chemical biosorption takes place between the biosorbent and zinc ions. The experimental data was also fitted well by the D–R isotherm model, as supported by a high determination coefficient (*R*^2^ = 0.967).

### 3.9. Impact of Temperature and Thermodynamic Modeling

**Figure 8:**
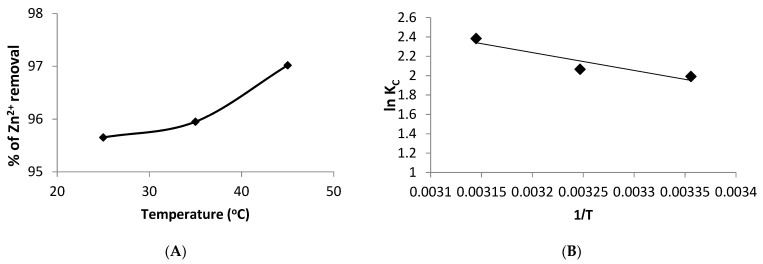
(**A**) Effect of temperature on the biosorption process, and (**B**) plot of ln*Kc* against *1/T* for the determination of thermodynamic parameters.

The influence of temperature (25, 35, and 45 °C) on Zn^2+^ ion biosorption was examined. Figure 8A demonstrated that increasing the temperature from 25 °C to 45 °C slightly enhanced the removal percentage of Zn^2+^ ions by *Spirulina* biomass from 95.6% to 97.02%, showing that the biosorption process was endothermic in nature. The increase in temperature may enhance the rate of metal diffusion over the exterior boundary layer and in the interior pores of the biosorbate particles because liquid viscosity lowers as the temperature constant increases [57].

This experiment was utilized to calculate the thermodynamic parameters, and the results are shown in Table 6.

The Gibbs’s free energy (Δ*G*°, kJ/mol), entropy (Δ*S*°, kJ/mol) and enthalpy (Δ*H*°, kJ/mol) values of the biosorption process were determined by the following equations:

(14) $$\Delta G{}^{\circ}=-RTlnK_{C}$$

(15) $$LnKc=\frac{\Delta S{}^{\circ}}{R}-\frac{\Delta H{}^{\circ}}{RT}\;\;\;\;\;\;\;\;\;\;\;\;\;\;$$

(16) ΔG°=ΔH°−T ΔS°

where *K_C_*: is related to equilibrium thermodynamic constant.

Table 6 summarizes the Δ*G°*, Δ*H°* and Δ*S°* values of the zinc biosorption by *Spirulina* biomass. The entropy and enthalpy were determined from the intercept and slope of the plot of ln*K_C_* against *1/T*, respectively, as indicated in Figure 8B.

The negative values of Δ*G*° suggested that the biosorption was thermodynamically spontaneous and feasible. Additionally, the reduction in Δ*G°* values as a temperature rises indicates that the biosorption is more feasible at higher temperatures.

The biosorption enthalpy (Δ*H°*) was determined to be 15.31 kJ/mol. In addition, the positive Δ*H°* value shows that the biosorption is endothermic, implying that sorption is enhanced at higher temperatures. The endothermic nature of biosorption was described by previous studies. In this respect, Safari and Ahmady-Asbchin [58] stated a decrease in Zn^2+^ ions biosorption onto *Fischerella ambigua* biomass with a rise in temperature. In addition, Zinicovscaia et al. [3] found that increasing the temperature from 293 to 323 K reduced the biosorption of Zn^2+^ ions by the dried biomass of *Spirulina platensis*. Lastly, the positive Δ*S°* value (0.068 kJ/mol) showed an enhanced degree of randomness at the solid–liquid interface [47].

### 3.10. Proposed Mechanism of Zn^2+^ Biosorption by S. platensis

SEM combined with EDX analysis revealed considerable changes following the biosorption process. The exchange and disappearance of some elements after biosorption process suggested that the biosorption of Zn^2+^ ions was caused by ion exchange. Furthermore, the *S. platensis* biomass has a point of zero charge of 6.5, implying that the algal surface has a positive charge at pH < pH_pzc_ and a negative charge at pH > pH_pzc_. Since the optimum pH for zinc ion biosorption onto the algal biomass is 7 (pH > pH_pzc_), so there is an electrostatic interaction between Zn^2+^ ions and the negatively charged functional groups on the algal surface, such as N−H, –OH, and –COOH. These functional groups that include nitrogen and/or oxygen aid in the biosorption process by formation of hydrogen bonds between the zinc ions and the algal surface, improving the Zn^2+^ biosorption process. The findings further proposed that the biosorption mechanism of Zn^2+^ is mediated by complexation of the functional groups on the surface of *S. platensis* with zinc ions via electrostatic attraction forces and ion exchange [59]. According to these findings, it can be concluded that the fundamental mechanism between the zinc ions and algal biomass is controlled and established by electrostatic interaction, formation of hydrogen bond and ion exchange.

### 3.11. Comparison of Zn^2+^ Biosorption Capacity

Table 7 shows a comparison of the maximum zinc adsorption capacity of several adsorbents. This study indicated that the maximum biosorption capacity of *Spirulina platensis* is much greater than that of the previously described adsorbents [3,60,61,62,63,64,65,66]. Thus, *Spirulina platensis* biomass might be regarded a viable low-cost biosorbent for removing zinc ions from wastewater.

## 4. Conclusions

Several industrial effluents include high quantities of heavy metals such as zinc, which cause major health and environmental hazards. Biosorption is a biotechnology strategy for heavy metal ion removal from polluted aquatic environments. The goal of this work is to apply a statistical design to improve parameters for maximal biosorption of zinc ions from the aqueous solutions by response surface methodology. The data demonstrated that RSM found to be an effective approach for determining the influence of process variables and their interactions on biosorption of Zn^2+^ ions onto the cyanobacterial alga *Spirulina platensis*. According to the quadratic model, a maximum removal of zinc ions of 97.9% was achieved by the optimal biosorption conditions such as 4.48 g/L of algal dose, pH of 6.62, and initial zinc concentration of 29.72 mg/L at a contact time of 60 min and 25 °C. The experimental data was correlated using three kinetic models such as pseudo-first, -second order, and intra-particle diffusion model, and the kinetic parameters were derived. The findings showed that biosorption process fitted well with the pseudo-second order kinetic model, implying that the adsorption mechanism is chemical in nature. The Langmuir and Dubinin–Radushkevich isotherm models suit well the experimental data. The thermodynamic parameters revealed that the biosorption process is spontaneous, endothermic and feasible at 298–323 K. FT-IR analysis of algal biomass showed the presence of methyl, phosphate, amine, amide, carboxyl, hydroxyl, and methylene groups, which are responsible for the biosorption process. Furthermore, the capacity of *Spirulina platensis* biomass to remove Zn^2+^ ions from the aqueous solutions is confirmed by the SEM and EDX analyses. An extra absorption peak related to Zn^2+^ ion was appeared by EDX analysis. The biosorption mechanism of zinc ion onto the *Spirulina platensis* biomass was controlled by electrostatic interaction, formation of hydrogen bond and ion exchange. Finally, it was concluded that the biomass of *Spirulina platensis* can efficiently remove Zn^2+^ ions from the solutions under RSM-optimized parameters.

## Figures and Tables

**Table 1:** Experimental design in terms of actual and coded variables of Zn^2+^ ions.

	Actual and Coded Values			Zinc Removal (%)	
Run Order	Algal Dose (g/L)	B: pH	C: Initial Zn^2+^ Conc. (mg/L)	Actual	Predicted
1	1(−1)	3(−1)	40(0)	75.00	76.42
2	5(+1)	3(−1)	40(0)	93.90	95.14
3	1(−1)	7(+1)	40(0)	98.58	97.70
4	5(+1)	7(+1)	40(0)	99.33	98.27
5	1(−1)	5(0)	20(−1)	97.30	93.78
6	5(+1)	5(0)	20(−1)	91.46	88.12
7	1(−1)	5(0)	60(+1)	70.00	70.01
8	5(+1)	5(0)	60(+1)	94.77	94.96
9	3(0)	3(−1)	20(−1)	94.24	94.67
10	3(0)	7(+1)	20(−1)	94.84	97.57
11	3(0)	3(−1)	60(+1)	80.00	76.90
12	3(0)	7(+1)	60(+1)	99.20	98.40
13–17 ^a^	3(0)	5(0)	40(0)	95.34	86.72

^a^ mean value of five center point assays.

**Table 2:** ANOVA for the response surface quadratic polynomial model.

Source of Variations	Sum of Squares	Degree of Freedom	Mean Sum of Squares	*F* Value	Probability
**Regression**	1143.3	7	163.3	6.70	0.005 *
**Residual**	219.3	9	24.4	-	-
**Lack of Fit**	55.7	5	11.1	0.27	0.91 **
**Pure Error**	163.5	4	40.9	-	-
**Correlation Total**	1362.6	16	-	-	-
**R^2^ =** 0.89	**Adjusted R^2^ =** 0.81	**Predicted R^2^ =** 0.70	**Adequate precision =** 8.4	**Mean =** 89.2	**% coefficient of variation =** 5.5

* Significant at *p* ˂ 0.05; ** Not significant at *p* > 0.05.

**Table 3:** ANOVA data for the coefficients of regression model for removal of Zn^2+^ ions.

Model Term	Coefficient Estimate	Degree of Freedom	Standard Error	*F* Value	*p*-Value Prob > F
**Intercept**	86.7	1	1.65	-	-
**A-algal dose**	4.82	1	1.75	7.64	0.038
**B-pH**	6.10	1	1.75	12.22	0.007
**C-Zn^2+^ conc.**	−4.23	1	1.75	5.88	0.022
**AB**	−4.54	1	2.47	3.38	0.099
**AC**	7.65	1	2.47	9.61	0.013
**BC**	4.65	1	2.47	3.55	0.092
**B^2^**	5.14	1	2.40	4.64	0.059

**Table 4:** Parameter values of the isotherm kinetic models for Zn^2+^ ions biosorbed onto *S. platensis* biomass.

Parameters		Values
**Experimental data**	***q_e_* (exp.) (mg g** **^−1^)**	19.60
**Pseudo-first order**	***q_e_* (cal.) (mg g** **^−1^)**	1.10
	***k*_1_ (min** **^−1^)**	0.066
	***R* ^2^**	0.816
**Pseudo-second order**	***q_e_* (cal.)(mg g** **^−1^)**	19.57
	***k*_2_ (gs mg** **^−1^ min** **^−1^)**	0.29
	***R* ^2^**	0.999
**Intra-particle diffusion**	***K_i_*** **(mgg^−1^ min^−0.5^)**	0.016
	***C_i_*** **(mg g^−1^)**	18.83
	***R*** **^2^**	0.382

**Table 5:** Parameter values of the isotherm models for Zn^2+^ ions biosorbed onto *S. platensis* biomass.

Isotherms	Parameters	Values
**Langmuir**	***q_max_* (mg g^−1^)**	50.7
	***b* (L mg^−1^)**	0.317
	***R_L_***	0.03–0.12
	***R* ^2^**	0.963
**Freundlich**	**1*/n***	0.68
	***K_f_* (L mg** **^−1^)**	11.54
	***R* ^2^**	0.945
**D-R**	***q_o_* (mg g^−1^)**	28.5
	***β* × 10^−7^ (mol^2^ J^−2^)**	2.0
	***E* (kJ mol^−1^)**	15.8
	***R* ^2^**	0.967

**Table 6:** Thermodynamic parameters for Zn^2+^ ions biosorbed onto *S. platensis* biomass.

Temperature (K)	Δ*G*° (kJ mol^−1^)	Δ*H*° (kJ mol^−1^)	Δ*S*° (kJ mol^−1^)	*R* ^2^
298	−4.93	15.31	0.068	0.874
308	−5.28			
318	−6.29			

**Table 7:** Maximum sorption capacity of Zn^2+^ ion by different sorbents.

Sorbents	*q_max_* (mg/g)	Reference
*Spirulina platensis*	7.1	[3]
*Raphidocelis subcapitata*	10.77	[60]
*Kappaphycus* sp.	16.78	[61]
Sugarcane bagasse	40.0	[62]
Coconut tree sawdust	23.81	[62]
Immobilized *Chlorella* sp.	28.5	[63]
Kaolinite	4.95	[64]
Immobilized *Chlorella vulgaris*	9.38	[65]
*Sargassum* sp.	1.914	[66]
*Spirulina platensis*	50.7	Present study

## Data Availability

Not applicable.
